# Supplementary material for: Host Transcriptome and Microbial Variation in Relation to Visceral Hyperalgesia
Source: Nutrients. 2025 Mar 6;17(5):921. doi: 10.3390/nu17050921 (PMC11902232; doi:10.3390/nu17050921)
Supplement: Supplementary file 1 [file nutrients-17-00921-s001.zip › nutrients-3492547-supplementary.pdf]

SUPPLEMENTARY TABLES

Table S1: Correlation of bacterial OTUs to IVP intensity.

OTU ID	Phylum	Class	Order	Family	*Genus*	*species*	r	p
881	Actinobacteria	Actinobacteria	Acidimicrobiales	CL500-29	*unclassified*		-0.64	0.005
311	Actinobacteria	Actinobacteria	Actinomycetales	Actinomycetaceae	*Actinomyces*		0.57	0.013
367	Actinobacteria	Actinobacteria	Actinomycetales	Brevibacteriaceae	*Brevibacterium*	*paucivorans*	-0.58	0.011
894	Actinobacteria	Actinobacteria	Actinomycetales	Cellulomonadaceae	*unclassified*		-0.49	0.039
960	Actinobacteria	Actinobacteria	Actinomycetales	Intrasporangiaceae	*unclassified*		-0.63	0.005
892	Actinobacteria	Actinobacteria	Actinomycetales	Microbacteriaceae	*unclassified*		-0.71	0.001
489	Actinobacteria	Actinobacteria	Actinomycetales	Micrococcaceae	*Arthrobacter*		-0.68	0.002
526	Actinobacteria	Actinobacteria	Actinomycetales	Micrococcaceae	*Arthrobacter*		-0.65	0.003
143	Actinobacteria	Actinobacteria	Actinomycetales	Micrococcaceae	*Arthrobacter*		-0.64	0.004
895	Actinobacteria	Actinobacteria	Actinomycetales	Micrococcaceae	*Arthrobacter*		-0.48	0.045
431	Actinobacteria	Actinobacteria	Actinomycetales	Micrococcaceae	*Rothia*	*aeria*	-0.59	0.009
141	Actinobacteria	Actinobacteria	Actinomycetales	Micrococcaceae	*Rothia*	*dentocariosa*	-0.60	0.009
194	Actinobacteria	Actinobacteria	Actinomycetales	Mycobacteriaceae	*Mycobacterium*		-0.65	0.003
821	Actinobacteria	Actinobacteria	Actinomycetales	Mycobacteriaceae	*Mycobacterium*		-0.62	0.006
383	Actinobacteria	Actinobacteria	Actinomycetales	Mycobacteriaceae	*Mycobacterium*		-0.55	0.017
195	Actinobacteria	Actinobacteria	Actinomycetales	Mycobacteriaceae	*Mycobacterium*		-0.50	0.034
481	Actinobacteria	Actinobacteria	Actinomycetales	Streptomycetaceae	*Streptomyces*		-0.77	2.E-04
896	Actinobacteria	Actinobacteria	Actinomycetales	Streptomycetaceae	*Streptomyces*		-0.70	0.001
436	Actinobacteria	Actinobacteria	Actinomycetales	Streptomycetaceae	*Streptomyces*		-0.67	0.002
488	Actinobacteria	Actinobacteria	Actinomycetales	Streptomycetaceae	*Streptomyces*		-0.63	0.005
893	Actinobacteria	Actinobacteria	Actinomycetales	Streptomycetaceae	*Streptomyces*		-0.60	0.008
959	Actinobacteria	Actinobacteria	Actinomycetales	Streptomycetaceae	*Streptomyces*		-0.56	0.016
891	Actinobacteria	Actinobacteria	Actinomycetales	Thermomonosporaceae	*Actinomadura*		-0.53	0.025
792	Actinobacteria	Actinobacteria	Actinomycetales	unclassified	*unclassified*		-0.66	0.003
63	Actinobacteria	Actinobacteria	Actinomycetales	unclassified	*unclassified*		-0.60	0.008
487	Actinobacteria	Actinobacteria	Actinomycetales	unclassified	*unclassified*		-0.60	0.009
527	Actinobacteria	Actinobacteria	Actinomycetales	unclassified	*unclassified*		-0.54	0.021
635	Bacteroidetes	Bacteroidia	Bacteroidales	Porphyromonadaceae	*Tannerella*	*forsythia*	0.54	0.022
147	Bacteroidetes	Bacteroidia	Bacteroidales	Porphyromonadaceae	*Tannerella*	*forsythia*	0.52	0.026
403	Bacteroidetes	Bacteroidia	Bacteroidales	Porphyromonadaceae	*Tannerella*	*forsythia*	0.48	0.044
1003	Bacteroidetes	Bacteroidia	Bacteroidales	Porphyromonadaceae	*Tannerella*		0.48	0.046
1017	Bacteroidetes	Bacteroidia	Bacteroidales	Porphyromonadaceae	*unclassified*		0.52	0.028
206	Bacteroidetes	Bacteroidia	Bacteroidales	Prevotellaceae	*Prevotella*		0.70	0.001
640	Bacteroidetes	Bacteroidia	Bacteroidales	Prevotellaceae	*Prevotella*		0.61	0.007
639	Bacteroidetes	Bacteroidia	Bacteroidales	Prevotellaceae	*Prevotella*		0.60	0.009
900	Bacteroidetes	Bacteroidia	Bacteroidales	Prevotellaceae	*Prevotella*		0.58	0.011
446	Bacteroidetes	Bacteroidia	Bacteroidales	Prevotellaceae	*Prevotella*		0.57	0.014
906	Bacteroidetes	Bacteroidia	Bacteroidales	Prevotellaceae	*Prevotella*		0.56	0.016
1008	Bacteroidetes	Bacteroidia	Bacteroidales	Prevotellaceae	*Prevotella*		0.51	0.029
901	Bacteroidetes	Bacteroidia	Bacteroidales	Prevotellaceae	*Prevotella*		0.51	0.030
655	Bacteroidetes	Bacteroidia	Bacteroidales	RikenellaceaeII	*unclassified*		-0.74	4.E-04
212	Bacteroidetes	Bacteroidia	Bacteroidales	RikenellaceaeII	*unclassified*		-0.71	0.001
439	Bacteroidetes	Bacteroidia	Bacteroidales	RikenellaceaeII	*unclassified*		-0.72	0.001
531	Bacteroidetes	Bacteroidia	Bacteroidales	RikenellaceaeII	*unclassified*		-0.70	0.001
532	Bacteroidetes	Bacteroidia	Bacteroidales	RikenellaceaeII	*unclassified*		-0.72	0.001
915	Bacteroidetes	Bacteroidia	Bacteroidales	RikenellaceaeII	*unclassified*		-0.68	0.002
78	Bacteroidetes	Bacteroidia	Bacteroidales	RikenellaceaeII	*unclassified*		-0.65	0.003
654	Bacteroidetes	Bacteroidia	Bacteroidales	RikenellaceaeII	*unclassified*		-0.59	0.010
77	Bacteroidetes	Bacteroidia	Bacteroidales	RikenellaceaeII	*unclassified*		-0.58	0.012
211	Bacteroidetes	Bacteroidia	Bacteroidales	RikenellaceaeII	*unclassified*		0.57	0.013
442	Bacteroidetes	Bacteroidia	Bacteroidales	RikenellaceaeII	*unclassified*		0.50	0.033
1027	Bacteroidetes	Bacteroidia	Bacteroidales	RikenellaceaeII	*unclassified*		-0.50	0.035
618	Bacteroidetes	Bacteroidia	Bacteroidales	RikenellaceaeII	*unclassified*		-0.47	0.047
450	Bacteroidetes	Flavobacteria	Flavobacteriales	Flavobacteriaceae	*Capnocytophaga*	*sputigena*	-0.48	0.042

Table S1: Continued…

OTU ID	Phylum	Class	Order	Family	*Genus*	*species*	r	p
849	Bacteroidetes	Flavobacteria	Flavobacteriales	Flavobacteriaceae	*Flavobacterium*		-0.49	0.039
407	Bacteroidetes	Flavobacteria	Flavobacteriales	Flavobacteriaceae	*Haloanella*		-0.52	0.029
406	Bacteroidetes	Flavobacteria	Flavobacteriales	Flavobacteriaceae	*unclassified*		-0.54	0.020
620	Bacteroidetes	unclassified	unclassified	unclassified	*unclassified*		0.60	0.009
221	Bacteroidetes	unclassified	unclassified	unclassified	*unclassified*		-0.54	0.022
1031	Elusimicrobia	Elusimicrobia	Elusimicrobiales	unclassified	*unclassified*		-0.63	0.005
558	Firmicutes	Bacilli	Bacillales	Bacillaceae	*Bacillus*		-0.81	5.E-05
793	Firmicutes	Bacilli	Bacillales	Bacillaceae	*Bacillus*		-0.79	8.E-05
65	Firmicutes	Bacilli	Bacillales	Bacillaceae	*Bacillus*		-0.70	0.001
1048	Firmicutes	Bacilli	Bacillales	Bacillaceae	*Bacillus*		-0.73	0.001
732	Firmicutes	Bacilli	Bacillales	Bacillaceae	*Bacillus*		-0.66	0.003
1043	Firmicutes	Bacilli	Bacillales	Bacillaceae	*Bacillus*		-0.63	0.005
1066	Firmicutes	Bacilli	Bacillales	Bacillaceae	*Bacillus*		-0.63	0.005
797	Firmicutes	Bacilli	Bacillales	Bacillaceae	*Bacillus*		-0.58	0.011
111	Firmicutes	Bacilli	Bacillales	Bacillaceae	*Bacillus*		-0.54	0.022
1063	Firmicutes	Bacilli	Bacillales	Bacillaceae	*Bacillus*		-0.53	0.023
963	Firmicutes	Bacilli	Bacillales	Staphylococcaceae	*Staphylococcus*	*epidermidis*	-0.58	0.012
452	Firmicutes	Bacilli	Bacillales	unclassified	*unclassified*		-0.83	2.E-05
99	Firmicutes	Bacilli	Gemellales	Gemellaceae	*Gemella*	*sanguinis*	-0.79	1.E-04
1062	Firmicutes	Bacilli	Gemellales	Gemellaceae	*Gemella*		-0.76	3.E-04
226	Firmicutes	Bacilli	Gemellales	Gemellaceae	*Gemella*		-0.72	0.001
831	Firmicutes	Bacilli	Gemellales	Gemellaceae	*Gemella*		-0.73	0.001
832	Firmicutes	Bacilli	Gemellales	Gemellaceae	*Gemella*		-0.73	0.001
32	Firmicutes	Bacilli	Gemellales	Gemellaceae	*Gemella*		-0.62	0.007
33	Firmicutes	Bacilli	Lactobacillales	Aerococcaceae	*Aerococcus*	*viridans*	-0.56	0.016
969	Firmicutes	Bacilli	Lactobacillales	Aerococcaceae	*Aerococcus*	*viridans*	-0.52	0.027
1044	Firmicutes	Bacilli	Lactobacillales	Enterococcaceae	*Enterococcus*		-0.52	0.026
966	Firmicutes	Bacilli	Lactobacillales	Enterococcaceae	*unclassified*		-0.61	0.007
922	Firmicutes	Bacilli	Lactobacillales	Streptococcaceae	*Streptococcus*		-0.73	0.001
126	Firmicutes	Bacilli	Lactobacillales	Streptococcaceae	*Streptococcus*		-0.61	0.007
497	Firmicutes	Bacilli	Lactobacillales	Streptococcaceae	*Streptococcus*		-0.58	0.012
621	Firmicutes	Bacilli	Lactobacillales	Streptococcaceae	*Streptococcus*		-0.58	0.012
623	Firmicutes	Bacilli	Lactobacillales	Streptococcaceae	*Streptococcus*		-0.56	0.016
967	Firmicutes	Bacilli	Lactobacillales	Streptococcaceae	*Streptococcus*		-0.52	0.027
333	Firmicutes	Bacilli	Lactobacillales	unclassified	*unclassified*		-0.60	0.009
564	Firmicutes	Clostridia	Clostridiales	Clostridiaceae	*Clostridium*		-0.66	0.003
1035	Firmicutes	Clostridia	Clostridiales	Clostridiaceae	*Clostridium*		0.56	0.017
132	Firmicutes	Clostridia	Clostridiales	Clostridiales Family XIII	*Incertae Sedis*		0.57	0.014
138	Firmicutes	Clostridia	Clostridiales	Lachnospiraceae	*Clostridium*		-0.81	5.E-05
66	Firmicutes	Clostridia	Clostridiales	Lachnospiraceae	*Clostridium*		-0.53	0.023
928	Firmicutes	Clostridia	Clostridiales	Lachnospiraceae	*unclassified*		-0.85	1.E-05
1047	Firmicutes	Clostridia	Clostridiales	Lachnospiraceae	*unclassified*		-0.79	1.E-04
246	Firmicutes	Clostridia	Clostridiales	Lachnospiraceae	*unclassified*		-0.74	4.E-04
60	Firmicutes	Clostridia	Clostridiales	Lachnospiraceae	*unclassified*		-0.70	0.001
151	Firmicutes	Clostridia	Clostridiales	Lachnospiraceae	*unclassified*		-0.73	0.001
323	Firmicutes	Clostridia	Clostridiales	Lachnospiraceae	*unclassified*		-0.72	0.001
563	Firmicutes	Clostridia	Clostridiales	Lachnospiraceae	*unclassified*		-0.71	0.001
597	Firmicutes	Clostridia	Clostridiales	Lachnospiraceae	*unclassified*		-0.71	0.001
615	Firmicutes	Clostridia	Clostridiales	Lachnospiraceae	*unclassified*		-0.70	0.001
625	Firmicutes	Clostridia	Clostridiales	Lachnospiraceae	*unclassified*		-0.69	0.001
788	Firmicutes	Clostridia	Clostridiales	Lachnospiraceae	*unclassified*		-0.69	0.001
1046	Firmicutes	Clostridia	Clostridiales	Lachnospiraceae	*unclassified*		-0.72	0.001
1049	Firmicutes	Clostridia	Clostridiales	Lachnospiraceae	*unclassified*		-0.73	0.001
1051	Firmicutes	Clostridia	Clostridiales	Lachnospiraceae	*unclassified*		-0.70	0.001

Table S1: Continued…

OTU ID	Phylum	Class	Order	Family	*Genus*	*species*	r	p
27	Firmicutes	Clostridia	Clostridiales	Lachnospiraceae	*unclassified*		-0.68	0.002
175	Firmicutes	Clostridia	Clostridiales	Lachnospiraceae	*unclassified*		-0.67	0.002
603	Firmicutes	Clostridia	Clostridiales	Lachnospiraceae	*unclassified*		-0.67	0.002
622	Firmicutes	Clostridia	Clostridiales	Lachnospiraceae	*unclassified*		-0.69	0.002
931	Firmicutes	Clostridia	Clostridiales	Lachnospiraceae	*unclassified*		-0.67	0.002
10	Firmicutes	Clostridia	Clostridiales	Lachnospiraceae	*unclassified*		-0.66	0.003
29	Firmicutes	Clostridia	Clostridiales	Lachnospiraceae	*unclassified*		-0.66	0.003
85	Firmicutes	Clostridia	Clostridiales	Lachnospiraceae	*unclassified*		-0.66	0.003
807	Firmicutes	Clostridia	Clostridiales	Lachnospiraceae	*unclassified*		-0.66	0.003
329	Firmicutes	Clostridia	Clostridiales	Lachnospiraceae	*unclassified*		-0.65	0.004
598	Firmicutes	Clostridia	Clostridiales	Lachnospiraceae	*unclassified*		-0.65	0.004
602	Firmicutes	Clostridia	Clostridiales	Lachnospiraceae	*unclassified*		-0.65	0.004
624	Firmicutes	Clostridia	Clostridiales	Lachnospiraceae	*unclassified*		-0.64	0.004
789	Firmicutes	Clostridia	Clostridiales	Lachnospiraceae	*unclassified*		-0.65	0.004
112	Firmicutes	Clostridia	Clostridiales	Lachnospiraceae	*unclassified*		-0.63	0.005
345	Firmicutes	Clostridia	Clostridiales	Lachnospiraceae	*unclassified*		0.63	0.005
28	Firmicutes	Clostridia	Clostridiales	Lachnospiraceae	*unclassified*		-0.61	0.007
925	Firmicutes	Clostridia	Clostridiales	Lachnospiraceae	*unclassified*		-0.59	0.009
1045	Firmicutes	Clostridia	Clostridiales	Lachnospiraceae	*unclassified*		-0.60	0.009
172	Firmicutes	Clostridia	Clostridiales	Lachnospiraceae	*unclassified*		-0.58	0.011
927	Firmicutes	Clostridia	Clostridiales	Lachnospiraceae	*unclassified*		-0.58	0.011
784	Firmicutes	Clostridia	Clostridiales	Lachnospiraceae	*unclassified*		-0.58	0.012
11	Firmicutes	Clostridia	Clostridiales	Lachnospiraceae	*unclassified*		-0.57	0.013
463	Firmicutes	Clostridia	Clostridiales	Lachnospiraceae	*unclassified*		-0.57	0.014
328	Firmicutes	Clostridia	Clostridiales	Lachnospiraceae	*unclassified*		-0.56	0.016
464	Firmicutes	Clostridia	Clostridiales	Lachnospiraceae	*unclassified*		0.56	0.016
466	Firmicutes	Clostridia	Clostridiales	Lachnospiraceae	*unclassified*		0.55	0.018
1054	Firmicutes	Clostridia	Clostridiales	Lachnospiraceae	*unclassified*		-0.55	0.018
238	Firmicutes	Clostridia	Clostridiales	Lachnospiraceae	*unclassified*		0.54	0.019
1052	Firmicutes	Clostridia	Clostridiales	Lachnospiraceae	*unclassified*		-0.54	0.021
462	Firmicutes	Clostridia	Clostridiales	Lachnospiraceae	*unclassified*		-0.53	0.024
707	Firmicutes	Clostridia	Clostridiales	Lachnospiraceae	*unclassified*		0.53	0.025
940	Firmicutes	Clostridia	Clostridiales	Lachnospiraceae	*unclassified*		0.53	0.025
783	Firmicutes	Clostridia	Clostridiales	Lachnospiraceae	*unclassified*		0.52	0.028
721	Firmicutes	Clostridia	Clostridiales	Lachnospiraceae	*unclassified*		0.51	0.029
665	Firmicutes	Clostridia	Clostridiales	Lachnospiraceae	*unclassified*		0.51	0.030
924	Firmicutes	Clostridia	Clostridiales	Lachnospiraceae	*unclassified*		-0.51	0.030
30	Firmicutes	Clostridia	Clostridiales	Lachnospiraceae	*unclassified*		-0.51	0.032
662	Firmicutes	Clostridia	Clostridiales	Lachnospiraceae	*unclassified*		-0.50	0.035
241	Firmicutes	Clostridia	Clostridiales	Lachnospiraceae	*unclassified*		0.50	0.036
705	Firmicutes	Clostridia	Clostridiales	Lachnospiraceae	*unclassified*		0.49	0.038
796	Firmicutes	Clostridia	Clostridiales	Lachnospiraceae	*unclassified*		-0.49	0.039
805	Firmicutes	Clostridia	Clostridiales	Lachnospiraceae	*unclassified*		-0.48	0.043
374	Firmicutes	Clostridia	Clostridiales	Lachnospiraceae	*unclassified*		0.48	0.046
58	Firmicutes	Clostridia	Clostridiales	Peptostreptococcaceae	*Peptostreptococcus*		0.50	0.034
767	Firmicutes	Clostridia	Clostridiales	Ruminococcaceae	*unclassified*		0.68	0.002
1050	Firmicutes	Clostridia	Clostridiales	Ruminococcaceae	*unclassified*		-0.65	0.004
748	Firmicutes	Clostridia	Clostridiales	Ruminococcaceae	*unclassified*		0.48	0.041
332	Firmicutes	Clostridia	Clostridiales	Ruminococcaceae	*unclassified*		-0.48	0.046
600	Firmicutes	Clostridia	Clostridiales	unclassified	*unclassified*		-0.66	0.003
467	Firmicutes	Clostridia	Clostridiales	unclassified	*unclassified*		0.60	0.008
457	Firmicutes	Clostridia	Clostridiales	unclassified	*unclassified*		-0.59	0.010
946	Firmicutes	Clostridia	Clostridiales	unclassified	*unclassified*		0.59	0.010
318	Firmicutes	Clostridia	Clostridiales	unclassified	*unclassified*		0.55	0.017

Table S1: Continued…

OTU ID	Phylum	Class	Order	Family	*Genus*	*species*	r	p
971	Firmicutes	Clostridia	Clostridiales	unclassified	*unclassified*		0.53	0.024
251	Firmicutes	Clostridia	Clostridiales	Veillonellaceae	*Dialister*	*invisus*	0.59	0.010
249	Firmicutes	Clostridia	Clostridiales	Veillonellaceae	*Dialister*	*invisus*	0.51	0.032
253	Firmicutes	Clostridia	Clostridiales	Veillonellaceae	*Dialister*	*pneumosintes*	0.49	0.039
862	Firmicutes	Clostridia	Clostridiales	Veillonellaceae	*unclassified*		0.50	0.037
390	Firmicutes	Clostridia	Clostridiales	Veillonellaceae	*Veillonella*	*dispar*	0.54	0.020
371	Firmicutes	Clostridia	Clostridiales	Veillonellaceae	*Veillonella*		0.54	0.021
373	Firmicutes	Clostridia	Clostridiales	Veillonellaceae	*Veillonella*		0.48	0.046
334	Firmicutes	unclassified	unclassified	unclassified	*unclassified*		-0.81	4.E-05
261	Firmicutes	unclassified	unclassified	unclassified	*unclassified*		-0.69	0.002
794	Firmicutes	unclassified	unclassified	unclassified	*unclassified*		-0.66	0.003
174	Firmicutes	unclassified	unclassified	unclassified	*unclassified*		-0.64	0.004
601	Firmicutes	unclassified	unclassified	unclassified	*unclassified*		-0.60	0.008
418	Fusobacteria	Fusobacteria	Fusobacteriales	Fusobacteriaceae	*Fusobacterium*		0.53	0.024
391	Fusobacteria	Fusobacteria	Fusobacteriales	Fusobacteriaceae	*Fusobacterium*		0.49	0.041
680	KSB1	Ucn15732	unclassified	unclassified	*unclassified*		0.53	0.023
681	NKB19	GN13	unclassified	unclassified	*unclassified*		-0.56	0.015
869	Proteobacteria	Alphaproteobacteria	Rhizobiales	Bradyrhizobiaceae	*unclassified*		-0.79	9.E-05
420	Proteobacteria	Alphaproteobacteria	Rhizobiales	Bradyrhizobiaceae	*unclassified*		-0.71	0.001
476	Proteobacteria	Alphaproteobacteria	Rhizobiales	Hyphomicrobiaceae	*Rhodoplanes*		-0.59	0.010
1133	Proteobacteria	Alphaproteobacteria	Rhizobiales	unclassified	*unclassified*		0.51	0.030
139	Proteobacteria	Alphaproteobacteria	Sphingomonadales	Erythrobacteraceae	*unclassified*		-0.62	0.006
268	Proteobacteria	Alphaproteobacteria	Sphingomonadales	Sphingomonadaceae	*Novosphingobium*		-0.50	0.035
14	Proteobacteria	Alphaproteobacteria	Sphingomonadales	Sphingomonadaceae	*Sphingomonas*	*echinoides*	-0.53	0.025
36	Proteobacteria	Alphaproteobacteria	Sphingomonadales	Sphingomonadaceae	*Sphingomonas*		-0.60	0.009
153	Proteobacteria	Alphaproteobacteria	Sphingomonadales	Sphingomonadaceae	*unclassified*		-0.54	0.020
747	Proteobacteria	Alphaproteobacteria	unclassified	unclassified	*unclassified*		0.53	0.024
1086	Proteobacteria	Alphaproteobacteria	unclassified	unclassified	*unclassified*		-0.48	0.045
158	Proteobacteria	Betaproteobacteria	Burkholderiales	Burkholderiaceae	*Ralstonia*		-0.62	0.006
46	Proteobacteria	Betaproteobacteria	Burkholderiales	Comamonadaceae	*Acidovorax*		-0.48	0.044
273	Proteobacteria	Betaproteobacteria	Burkholderiales	Oxalobacteraceae	*unclassified*		-0.48	0.042
1091	Proteobacteria	Deltaproteobacteria	Syntrophobacterales	Desulfobacteraceae	*Desulfobacterium*		-0.71	0.001
608	Proteobacteria	Deltaproteobacteria	Syntrophobacterales	Desulfobacteraceae	*unclassified*		-0.66	0.003
121	Proteobacteria	Epsilonproteobacteria	Campylobacterales	Campylobacteraceae	*Campylobacter*	*gracilis*	0.51	0.031
837	Proteobacteria	Epsilonproteobacteria	Campylobacterales	Campylobacteraceae	*Campylobacter*		0.53	0.024
16	Proteobacteria	Gammaproteobacteria	Alteromonadales	Chromatiaceae	*Rheinheimera*		-0.53	0.022
569	Proteobacteria	Gammaproteobacteria	Oceanospirillales	Halomonadaceae	*Candidatus Portiera*	*unclassified*	-0.58	0.011
321	Proteobacteria	Gammaproteobacteria	Oceanospirillales	unclassified	*unclassified*		-0.63	0.005
1076	Proteobacteria	Gammaproteobacteria	Oceanospirillales	unclassified	*unclassified*		-0.49	0.039
37	Proteobacteria	Gammaproteobacteria	Pseudomonadales	Moraxellaceae	*Acinetobacter*	*johnsonii*	-0.71	0.001
281	Proteobacteria	Gammaproteobacteria	Pseudomonadales	Moraxellaceae	*Acinetobacter*	*johnsonii*	-0.70	0.001
617	Proteobacteria	Gammaproteobacteria	Pseudomonadales	Moraxellaceae	*Acinetobacter*	*johnsonii*	-0.65	0.003
76	Proteobacteria	Gammaproteobacteria	Pseudomonadales	Moraxellaceae	*Acinetobacter*	*johnsonii*	-0.62	0.006
38	Proteobacteria	Gammaproteobacteria	Pseudomonadales	Moraxellaceae	*Acinetobacter*	*johnsonii*	-0.60	0.008
701	Proteobacteria	Gammaproteobacteria	Pseudomonadales	Moraxellaceae	*Acinetobacter*	*johnsonii*	-0.60	0.008
18	Proteobacteria	Gammaproteobacteria	Pseudomonadales	Moraxellaceae	*Acinetobacter*	*venetianus*	-0.65	0.004
279	Proteobacteria	Gammaproteobacteria	Pseudomonadales	Moraxellaceae	*Acinetobacter*	*venetianus*	-0.48	0.043
795	Proteobacteria	Gammaproteobacteria	Pseudomonadales	Moraxellaceae	*Acinetobacter*		-0.78	1.E-04
17	Proteobacteria	Gammaproteobacteria	Pseudomonadales	Moraxellaceae	*Acinetobacter*		-0.68	0.002
48	Proteobacteria	Gammaproteobacteria	Pseudomonadales	Moraxellaceae	*Acinetobacter*		-0.67	0.002
75	Proteobacteria	Gammaproteobacteria	Pseudomonadales	Moraxellaceae	*Acinetobacter*		-0.69	0.002
878	Proteobacteria	Gammaproteobacteria	Pseudomonadales	Moraxellaceae	*Acinetobacter*		-0.59	0.010
981	Proteobacteria	Gammaproteobacteria	Pseudomonadales	Moraxellaceae	*Acinetobacter*		-0.57	0.013
522	Proteobacteria	Gammaproteobacteria	Pseudomonadales	Moraxellaceae	*Acinetobacter*		-0.54	0.020

Table S1: Continued…

OTU ID	Phylum	Class	Order	Family	*Genus*	*species*	r	p
982	Proteobacteria	Gammaproteobacteria	Pseudomonadales	Moraxellaceae	*Acinetobacter*		-0.54	0.021
1084	Proteobacteria	Gammaproteobacteria	Pseudomonadales	Moraxellaceae	*Moraxella*		-0.58	0.011
424	Proteobacteria	Gammaproteobacteria	Pseudomonadales	Moraxellaceae	*Moraxella*		-0.54	0.020
325	Proteobacteria	Gammaproteobacteria	Pseudomonadales	Pseudomonadaceae	*Pseudomonas*		-0.72	0.001
506	Proteobacteria	Gammaproteobacteria	Pseudomonadales	Pseudomonadaceae	*Pseudomonas*		-0.71	0.001
591	Proteobacteria	Gammaproteobacteria	Pseudomonadales	Pseudomonadaceae	*Pseudomonas*		-0.67	0.002
1096	Proteobacteria	Gammaproteobacteria	Pseudomonadales	Pseudomonadaceae	*Pseudomonas*		-0.66	0.003
90	Proteobacteria	Gammaproteobacteria	Pseudomonadales	Pseudomonadaceae	*Pseudomonas*		-0.64	0.004
1094	Proteobacteria	Gammaproteobacteria	Pseudomonadales	Pseudomonadaceae	*Pseudomonas*		-0.64	0.004
91	Proteobacteria	Gammaproteobacteria	Pseudomonadales	Pseudomonadaceae	*Pseudomonas*		-0.63	0.005
26	Proteobacteria	Gammaproteobacteria	Pseudomonadales	Pseudomonadaceae	*Pseudomonas*		-0.62	0.006
1095	Proteobacteria	Gammaproteobacteria	Pseudomonadales	Pseudomonadaceae	*Pseudomonas*		-0.62	0.007
596	Proteobacteria	Gammaproteobacteria	Pseudomonadales	Pseudomonadaceae	*Pseudomonas*		-0.59	0.010
987	Proteobacteria	Gammaproteobacteria	Pseudomonadales	Pseudomonadaceae	*Pseudomonas*		-0.52	0.027
592	Proteobacteria	Gammaproteobacteria	unclassified	unclassified	*unclassified*		-0.65	0.004
1089	Proteobacteria	Gammaproteobacteria	unclassified	unclassified	*unclassified*		-0.62	0.006
806	Proteobacteria	Gammaproteobacteria	unclassified	unclassified	*unclassified*		-0.59	0.010
568	Proteobacteria	Gammaproteobacteria	unclassified	unclassified	*unclassified*		-0.58	0.011
980	Proteobacteria	Gammaproteobacteria	unclassified	unclassified	*unclassified*		-0.56	0.015
782	Proteobacteria	Gammaproteobacteria	unclassified	unclassified	*unclassified*		0.55	0.018
280	Proteobacteria	Gammaproteobacteria	Xanthomonadales	Xanthomonadaceae	*Xanthomonas*	*retroflexus*	-0.49	0.040
1085	Proteobacteria	unclassified	unclassified	unclassified	*unclassified*		-0.70	0.001
1092	Proteobacteria	unclassified	unclassified	unclassified	*unclassified*		-0.73	0.001
749	Proteobacteria	unclassified	unclassified	unclassified	*unclassified*		0.57	0.013
1077	Proteobacteria	unclassified	unclassified	unclassified	*unclassified*		0.55	0.018
118	Proteobacteria	unclassified	unclassified	unclassified	*unclassified*		-0.53	0.024
694	Proteobacteria	unclassified	unclassified	unclassified	*unclassified*		-0.52	0.025
778	Proteobacteria	unclassified	unclassified	unclassified	*unclassified*		0.51	0.030
791	Proteobacteria	unclassified	unclassified	unclassified	*unclassified*		-0.51	0.031
804	Proteobacteria	unclassified	unclassified	unclassified	*unclassified*		-0.51	0.031
567	Proteobacteria	unclassified	unclassified	unclassified	*unclassified*		-0.50	0.035
286	Spirochaetes	Spirochaetes	Spirochaetales	Spirochaetaceae	*Treponema*	*socranskii*	0.53	0.024
697	Spirochaetes	Spirochaetes	Spirochaetales	Spirochaetaceae	*Treponema*		0.58	0.012
595	Tenericutes	Erysipelotrichi	Erysipelotrichales	Erysipelotrichaceae	*Bulleidia*		0.55	0.019
295	Tenericutes	Erysipelotrichi	Erysipelotrichales	Erysipelotrichaceae	*Eubacterium*	*biforme*	0.51	0.029
129	Tenericutes	Erysipelotrichi	Erysipelotrichales	Erysipelotrichaceae	*Solobacterium*	*moorei*	0.52	0.026
296	Tenericutes	Erysipelotrichi	Erysipelotrichales	Erysipelotrichaceae	*Solobacterium*	*moorei*	0.49	0.039
841	Tenericutes	Mollicutes	Acholeplasmatales	Acholeplasmataceae	*Candidatus Phytoplasma*	*unclassified*	-0.53	0.023
998	Tenericutes	Mollicutes	Mycoplasmatales	Mycoplasmataceae	*Mycoplasma*	*hominis*	0.72	0.001
298	Tenericutes	Mollicutes	Mycoplasmatales	Mycoplasmataceae	*Mycoplasma*		0.47	0.049
1136	Tenericutes	Mollicutes	unclassified	unclassified	*unclassified*		0.56	0.017
953	TM7	TM7-3	I025	Rs-045	*unclassified*		0.50	0.035

Table S2: Table of bacteria whose expression were highly significantly correlated to IVP intensity. All correlations were negative.

Phylum	Class	Order	Family	Genus	species	r2	p
Firmicutes	Clostridia	Clostridiales	Lachnospiraceae	unclassified	unclassified	0.72	0.00001
Firmicutes	Bacilli	Bacillales	unclassified	unclassified	unclassified	0.68	0.00002
Firmicutes	unclassified	unclassified	unclassified	unclassified	unclassified	0.66	0.00004
Firmicutes	Clostridia	Clostridiales	Lachnospiraceae	Clostridium	unclassified	0.65	0.00005
Firmicutes	Bacilli	Bacillales	Bacillaceae	Bacillus	unclassified	0.65	0.00005
Firmicutes	Bacilli	Bacillales	Bacillaceae	Bacillus	unclassified	0.63	0.00008
Proteobacteria	Alphaproteobacteria	Rhizobiales	Bradyrhizobiaceae	unclassified	unclassified	0.63	0.00009

Table S3: Correlation of peripherally expressed genes to IVP intensity.

ENTREZ ID	Gene Synbol	r	p	ENTREZ ID	Gene Synbol	r	p	ENTREZ ID	Gene Synbol	r	p
59	ACTA2	-0.56	0.009	2661	GDF9	0.62	0.003	6297	SALL2	-0.48	0.029
90	ACVR1	0.54	0.011	2786	GNG4	0.53	0.012	6304	SATB1	-0.44	0.043
130	ADH6	0.55	0.010	2806	GOT2	-0.44	0.045	6331	SCN5A	0.45	0.043
258	AMBN	0.45	0.039	2810	SFN	0.50	0.021	6345	SRL	-0.54	0.011
301	ANXA1	0.44	0.047	2833	CXCR3	-0.55	0.010	6373	CXCL11	0.60	0.004
319	APOF	-0.55	0.010	2835	GPR12	0.46	0.037	6492	SIM1	0.50	0.021
341	APOC1	0.43	0.049	2843	GPR20	0.44	0.043	6512	SLC1A7	0.54	0.011
362	AQP5	0.58	0.006	2888	GRB14	-0.47	0.033	6523	SLC5A1	0.46	0.035
509	ATP5C1	0.44	0.045	2909	ARHGAP35	-0.49	0.024	6530	SLC6A2	0.44	0.044
567	B2M	0.51	0.019	2921	CXCL3	0.54	0.011	6559	SLC12A3	-0.56	0.009
599	BCL2L2	0.44	0.046	2947	GSTM3	-0.52	0.015	6579	SLCO1A2	-0.54	0.012
627	BDNF	-0.47	0.030	2948	GSTM4	-0.50	0.021	6610	SMPD2	0.45	0.039
631	BFSP1	0.65	0.002	2957	GTF2A1	-0.50	0.022	6657	SOX2	0.48	0.028
651	BMP3	-0.47	0.031	3005	H1F0	0.49	0.023	6702	SPRR2C	0.69	0.001
659	BMPR2	-0.57	0.007	3204	HOXA7	-0.44	0.044	6711	SPTBN1	-0.44	0.045
733	C8G	0.46	0.037	3215	HOXB5	0.50	0.021	6725	SRMS	0.45	0.043
757	TMEM50B	0.44	0.046	3222	HOXC5	0.46	0.036	6734	SRPR	-0.45	0.040
858	CAV2	-0.47	0.032	3223	HOXC6	0.52	0.016	6855	SYP	0.48	0.028
899	CCNF	-0.51	0.018	3239	HOXD13	-0.44	0.046	6942	TCF20	-0.49	0.025
988	CDC5L	-0.45	0.040	3354	HTR1E	0.51	0.017	7004	TEAD4	0.45	0.040
998	CDC42	0.48	0.027	3362	HTR6	0.47	0.033	7007	TECTA	0.45	0.041
1009	CDH11	0.54	0.012	3443	IFNA6	0.59	0.004	7067	THRA	-0.46	0.035
1010	CDH12	0.47	0.034	3449	IFNA16	0.53	0.014	7092	TLL1	0.49	0.024
1129	CHRM2	0.55	0.009	3575	IL7R	-0.50	0.021	7125	TNNC2	-0.43	0.049
1234	CCR5	-0.47	0.030	3595	IL12RB2	-0.56	0.008	7201	TRHR	0.57	0.007
1237	CCR8	0.44	0.046	3641	INSL4	0.47	0.031	7224	TRPC5	-0.51	0.018
1268	CNR1	0.43	0.049	3655	ITGA6	-0.54	0.011	7267	TTC3	-0.46	0.035
1271	CNTFR	0.47	0.031	3656	IRAK2	-0.53	0.014	7280	TUBB2A	-0.52	0.015
1360	CPB1	0.47	0.032	3716	JAK1	-0.49	0.025	7301	TYRO3	-0.46	0.037
1555	CYP2B6	0.46	0.036	3745	KCNB1	0.49	0.026	7430	EZR	-0.49	0.025
1559	CYP2C9	0.50	0.020	3778	KCNMA1	-0.52	0.017	7436	VLDLR	-0.45	0.038
1592	CYP26A1	0.55	0.010	4869	NPM1	-0.45	0.039	7498	XDH	0.51	0.017
1620	BRINP1	0.48	0.029	5069	PAPPA	-0.49	0.025	7544	ZFY	0.59	0.005
1728	NQO1	0.55	0.010	5179	PENK	0.43	0.050	7586	ZKSCAN1	-0.55	0.010
1893	ECM1	0.65	0.001	5295	PIK3R1	-0.44	0.047	7587	ZNF37A	-0.50	0.021
2064	ERBB2	0.51	0.017	5319	PLA2G1B	0.48	0.029	7704	ZBTB16	-0.47	0.031
2069	EREG	0.44	0.044	5322	PLA2G5	0.64	0.002	7711	ZNF155	0.73	0.001
2239	GPC4	0.58	0.006	5346	PLIN1	0.46	0.035	7798	LUZP1	-0.45	0.039
2297	FOXD1	0.49	0.025	5376	PMP22	-0.54	0.012	8019	BRD3	-0.52	0.017
2317	FLNB	-0.50	0.021	5649	RELN	0.44	0.047	8031	NCOA4	0.65	0.001
2494	NR5A2	0.53	0.013	5650	KLK7	0.48	0.028	8038	ADAM12	-0.49	0.025
2516	NR5A1	-0.51	0.018	5802	PTPRS	-0.50	0.022	8332	HIST1H2AL	-0.49	0.024
2532	ACKR1	-0.50	0.022	5830	PEX5	0.44	0.048	8390	OR1G1	0.57	0.007
2554	GABRA1	0.44	0.046	5978	REST	-0.58	0.005	8470	SORBS2	0.46	0.038
2570	GABRR2	0.57	0.007	6185	RPN2	-0.56	0.008	8482	SEMA7A	-0.43	0.050
2595	GANC	-0.56	0.008	6240	RRM1	-0.45	0.043	8495	PPFIBP2	-0.46	0.037

Table S3: Continued…

ENTREZ ID	Gene Synbol	r	p	ENTREZ ID	Gene Synbol	r	p	ENTREZ ID	Gene Synbol	r	p
8506	CNTNAP1	0.44	0.048	11148	HHLA2	-0.55	0.010	51280	GOLM1	0.80	0.001
8513	LIPF	0.46	0.038	11170	FAM107A	0.54	0.012	51305	KCNK9	0.49	0.024
8534	CHST1	0.45	0.042	11198	SUPT16H	-0.44	0.047	51523	CXXC5	-0.51	0.019
8557	TCAP	0.53	0.013	11212	PROSC	-0.50	0.020	51594	NBAS	-0.47	0.030
8602	NOP14	-0.45	0.041	11226	GALNT6	-0.49	0.026	51639	SF3B6	0.44	0.045
8615	USO1	-0.48	0.027	11329	STK38	0.46	0.035	51667	NUB1	-0.48	0.029
8661	EIF3A	-0.49	0.024	22835	ZFP30	0.54	0.011	51700	CYB5R2	-0.45	0.039
8829	NRP1	-0.51	0.017	23028	KDM1A	-0.52	0.015	51701	NLK	-0.54	0.011
8840	WISP1	0.52	0.015	23099	ZBTB43	-0.46	0.038	51705	EMCN	0.61	0.003
8846	ALKBH1	0.44	0.046	23194	FBXL7	0.46	0.036	51773	RSF1	-0.44	0.045
8932	MBD2	0.46	0.034	23224	SYNE2	-0.47	0.031	53919	SLCO1C1	0.44	0.048
8997	KALRN	0.52	0.015	23247	KIAA0556	0.46	0.034	54429	TAS2R5	0.55	0.010
9001	HAP1	0.46	0.034	23361	ZNF629	-0.55	0.010	54457	TAF7L	0.47	0.032
9063	PIAS2	-0.46	0.035	23384	SPECC1L	-0.47	0.032	54541	DDIT4	-0.47	0.032
9126	SMC3	-0.47	0.032	23468	CBX5	-0.56	0.008	54545	MTMR12	0.52	0.015
9162	DGKI	-0.45	0.042	23547	LILRA4	-0.47	0.030	54738	FEV	0.49	0.023
9270	ITGB1BP1	-0.43	0.049	23582	CCNDBP1	0.47	0.030	54756	IL17RD	0.43	0.049
9355	LHX2	-0.45	0.042	23598	PATZ1	-0.52	0.015	54763	ROPN1	-0.54	0.011
9374	PPT2	-0.50	0.020	23613	ZMYND8	-0.54	0.011	54876	DCAF16	-0.44	0.044
9397	NMT2	-0.46	0.036	23627	PRND	0.48	0.027	55065	SLC52A1	0.45	0.043
9731	CEP104	-0.50	0.023	23769	FLRT1	-0.50	0.022	55117	SLC6A15	0.50	0.021
9739	SETD1A	-0.44	0.047	25831	HECTD1	-0.48	0.027	55137	FIGN	0.46	0.036
9851	KIAA0753	0.49	0.023	25888	ZNF473	-0.44	0.048	55226	NAT10	-0.45	0.042
9967	THRAP3	-0.46	0.037	25927	CNRIP1	-0.46	0.037	55329	MNS1	0.44	0.047
9978	RBX1	0.45	0.043	25960	GPR124	-0.52	0.016	55422	ZNF331	-0.46	0.038
10000	AKT3	-0.54	0.011	26070	DKFZP434K028	0.46	0.034	55540	IL17RB	0.49	0.023
10109	ARPC2	0.50	0.021	26188	OR1C1	0.62	0.003	55567	DNAH3	0.46	0.034
10128	LRPPRC	-0.47	0.032	26212	OR2B6	0.69	0.001	55640	FLVCR2	0.52	0.016
10141	C4orf6	0.49	0.024	26219	OR1J4	0.53	0.014	55661	DDX27	-0.46	0.036
10161	LPAR6	0.45	0.039	26227	PHGDH	-0.45	0.040	55870	ASH1L	-0.44	0.047
10238	DCAF7	-0.44	0.045	26692	OR2W1	0.52	0.015	55904	KMT2E	-0.51	0.018
10268	RAMP3	0.47	0.032	26998	FETUB	-0.48	0.027	56122	PCDHB14	0.51	0.018
10319	LAMC3	0.47	0.031	27293	SMPDL3B	0.51	0.019	56158	TEX12	-0.65	0.002
10344	CCL26	0.56	0.008	27300	ZNF544	-0.50	0.020	56252	YLPM1	-0.46	0.035
10350	ABCA9	0.60	0.004	27429	HTRA2	0.43	0.049	56287	GKN1	-0.47	0.032
10482	NXF1	0.48	0.027	28646	TRAV36DV7	0.49	0.024	56547	MMP26	-0.44	0.048
10518	CIB2	-0.46	0.035	28964	GIT1	-0.44	0.047	56833	SLAMF8	-0.45	0.040
10520	ZNF211	0.54	0.012	29114	TAGLN3	0.44	0.046	57062	DDX24	-0.55	0.010
10529	NEBL	0.48	0.027	29841	GRHL1	-0.44	0.046	57109	REXO4	-0.44	0.044
10584	COLEC10	0.51	0.019	29843	SENP1	0.47	0.031	57167	SALL4	0.44	0.047
10693	CCT6B	0.49	0.023	29993	PACSIN1	-0.45	0.042	57168	ASPHD2	-0.45	0.039
10758	TRAF3IP2	-0.46	0.034	29999	FSCN3	0.66	0.001	57212	TP73-AS1	-0.44	0.048
10825	NEU3	-0.45	0.040	50509	COL5A3	-0.43	0.049	57470	LRRC47	-0.45	0.040
10895	PPBPP2	0.47	0.032	50613	UBQLN3	0.54	0.012	57479	PRR12	-0.48	0.029
10940	POP1	-0.50	0.020	50615	IL21R	-0.44	0.044	57529	RGAG1	0.49	0.026
11009	IL24	-0.44	0.045	50652	PCA3	0.56	0.008	57530	CGN	0.51	0.019

Table S3: Continued…

ENTREZ ID	Gene Synbol	r	p	ENTREZ ID	Gene Synbol	r	p	ENTREZ ID	Gene Synbol	r	p
57583	TMEM181	0.43	0.049	84623	KIRREL3	-0.49	0.023	143279	HECTD2	0.47	0.033
57599	WDR48	0.44	0.044	84666	RETNLB	0.63	0.002	143903	LAYN	-0.70	0.001
57611	ISLR2	0.47	0.031	84667	HES7	0.43	0.049	144360	LINC00477	0.52	0.017
57685	CACHD1	-0.49	0.025	84792	FAM220A	0.46	0.037	146198	ZFP90	-0.49	0.023
57705	WDFY4	-0.54	0.011	84850	GLIS3-AS1	-0.47	0.033	146330	FBXL16	-0.47	0.031
57835	SLC4A5	-0.48	0.029	84870	RSPO3	0.44	0.045	146336	SSTR5-AS1	0.44	0.048
58477	SRPRB	-0.44	0.047	84996	URB1-AS1	0.48	0.028	146456	TMED6	0.50	0.021
59339	PLEKHA2	-0.54	0.011	89883	OR6W1P	-0.46	0.034	147646	C19orf84	0.54	0.011
60493	FASTKD5	-0.44	0.048	90167	FRMD7	0.44	0.048	147923	ZNF420	-0.46	0.037
60680	CELF5	0.57	0.007	90523	MLIP	0.57	0.007	148109	FAM187B	-0.43	0.049
63923	TNN	0.48	0.028	91368	CDKN2AIPNL	0.59	0.005	148327	CREB3L4	-0.52	0.017
63925	ZNF335	0.44	0.048	91689	SMDT1	-0.43	0.049	149373	LOC149373	0.50	0.022
63926	ANKEF1	-0.65	0.001	92211	CDHR1	-0.45	0.041	150197	LINC00896	0.55	0.009
64167	ERAP2	0.43	0.050	92304	SCGB3A1	0.44	0.044	150468	CKAP2L	0.44	0.047
65009	NDRG4	-0.44	0.045	92745	SLC38A5	-0.44	0.048	151194	METTL21A	0.52	0.015
79022	TMEM106C	0.47	0.033	93349	SP140L	-0.44	0.045	151531	UPP2	0.56	0.008
79066	METTL16	-0.50	0.021	93517	SDR42E1	-0.60	0.004	151877	MAGI1-IT1	0.46	0.034
79068	FTO	-0.48	0.028	94234	FOXQ1	0.50	0.020	152118	C3orf79	-0.56	0.008
79365	BHLHE41	-0.44	0.045	103910	MYL12B	0.63	0.002	153020	RASGEF1B	-0.44	0.044
79583	TMEM231	0.48	0.029	114609	TIRAP	0.45	0.039	154442	BVES-AS1	0.59	0.005
79600	TCTN1	0.43	0.049	115948	CCDC151	-0.44	0.044	154796	AMOT	-0.44	0.048
79723	SUV39H2	-0.46	0.037	116379	IL22RA2	0.45	0.042	155054	ZNF425	0.53	0.013
79746	ECHDC3	-0.45	0.042	117194	MRGPRX2	0.47	0.034	157247	MGC27345	-0.52	0.016
79747	ADGB	-0.62	0.003	117195	MRGPRX3	0.45	0.042	157273	LOC157273	0.65	0.001
79869	CPSF7	0.50	0.020	119391	GSTO2	0.57	0.007	159162	RBMY2FP	0.51	0.018
79896	THNSL1	-0.59	0.005	121256	TMEM132D	0.55	0.010	163778	SPRR4	0.45	0.042
79915	ATAD5	-0.57	0.007	121340	SP7	0.47	0.033	165530	CLEC4F	0.45	0.039
79949	PLEKHS1	-0.47	0.032	122616	C14orf79	0.50	0.021	165679	SPTSSB	-0.46	0.038
79991	OBFC1	-0.47	0.033	122809	SOCS4	0.43	0.049	166815	TIGD2	-0.45	0.039
80024	SLC8B1	0.54	0.012	122961	ISCA2	0.45	0.039	166863	RBM46	0.45	0.040
80198	MUS81	0.44	0.046	124359	CDYL2	0.52	0.017	168002	DACT2	0.52	0.016
80263	TRIM45	0.49	0.024	124540	MSI2	-0.44	0.048	168975	CNBD1	0.66	0.001
80309	SPHKAP	0.44	0.048	126549	ANKLE1	-0.45	0.041	169026	SLC30A8	0.48	0.027
80755	AARSD1	0.47	0.033	128859	BPIFB6	0.62	0.003	170850	KCNG3	0.51	0.019
80831	APOL5	0.44	0.047	129790	C7orf13	-0.46	0.038	192683	SCAMP5	-0.46	0.037
81615	TMEM163	-0.54	0.011	130916	MTERF4	-0.47	0.032	197257	LDHD	0.51	0.019
83856	FSD1L	-0.49	0.025	131450	CD200R1	-0.47	0.031	201516	ZSCAN4	0.50	0.020
83858	ATAD3B	0.45	0.043	133558	MROH2B	0.46	0.036	204801	NLRP11	-0.45	0.043
83860	TAF3	-0.44	0.048	133923	ZNF474	0.45	0.040	219578	ZNF804B	0.60	0.004
83894	TTC29	-0.62	0.003	136051	ZNF786	-0.44	0.045	221458	KIF6	0.48	0.027
84141	EVA1A	0.44	0.044	136227	COL26A1	0.53	0.014	221504	ZBTB9	-0.47	0.031
84181	CHD6	-0.49	0.025	136895	C7orf31	-0.45	0.043	221692	PHACTR1	-0.46	0.037
84245	MRI1	0.49	0.026	137964	AGPAT6	-0.47	0.032	221756	SERPINB9P1	-0.46	0.035
84260	TCHP	0.46	0.034	139420	SMEK3P	0.50	0.021	245936	DEFB123	0.48	0.029
84313	VPS25	0.44	0.048	139728	PNCK	0.46	0.037	252839	TMEM9	0.53	0.013
84318	CCDC77	-0.48	0.027	142680	SLC34A3	0.48	0.027	253152	EPHX4	0.44	0.048

Table S3: Continued…

ENTREZ ID	Gene Synbol	r	p	ENTREZ ID	Gene Synbol	r	p	ENTREZ ID	Gene Synbol	r	p
253461	ZBTB38	-0.45	0.041	387066	SNHG5	-0.48	0.030	100652856	LINC00408	-0.63	0.002
253559	CADM2	0.52	0.015	387082	SUMO4	0.51	0.018	100652931	LOC100652931	0.68	0.001
253639	ZNF620	-0.44	0.049	387758	FIBIN	0.46	0.036				
255025	LINC00879	0.47	0.031	642394	ADARB2-AS1	0.53	0.013				
255119	C4orf22	-0.44	0.046	642559	POU5F1P3	-0.44	0.046				
255193	CSNK1G2-AS1	0.46	0.038	643977	FLJ32255	-0.45	0.039				
255352	CFAP46	0.45	0.042	647288	CTAGE11P	0.49	0.025				
256051	ZNF549	-0.44	0.048	652995	UCA1	-0.45	0.040				
256227	STEAP1B	-0.45	0.040	677769	SCARNA17	-0.56	0.008				
256714	MAP7D2	-0.52	0.015	727944	LOC727944	0.55	0.010				
259234	DSCR10	0.44	0.046	728052	LOC728052	0.70	0.001				
259249	MRGPRX1	0.44	0.047	728485	LOC728485	0.46	0.034				
260429	PRSS33	-0.45	0.039	729085	FAM198A	0.50	0.022				
260434	PYDC1	0.45	0.042	729420	LMO7DN	0.48	0.029				
261734	NPHP4	0.52	0.016	780776	TVP23A	-0.44	0.049				
266675	BEST4	0.52	0.017	100101938	ANKRD26P3	0.46	0.034				
266917	D21S2088E	0.59	0.005	100124700	HOTAIR	0.49	0.025				
283847	CCDC79	0.44	0.048	100129271	C1orf68	0.45	0.040				
283902	HCCAT5	0.44	0.046	100129662	FGF13-AS1	-0.56	0.009				
284023	LOC284023	-0.51	0.019	100134713	NDUFB2-AS1	-0.44	0.044				
284257	BOD1L2	0.49	0.025	100170229	SRRM5	0.56	0.008				
284406	ZFP82	-0.45	0.042	100287765	LINC00630	0.45	0.042				
284900	TTC28-AS1	0.53	0.013	100288637	LOC100288637	-0.47	0.032				
285577	LINC01019	0.45	0.041	100289255	LINC00675	0.44	0.044				
285740	LOC285740	0.49	0.023	100316868	HOTTIP	0.48	0.028				
285761	DCBLD1	-0.52	0.015	100505491	LOC100505491	0.51	0.018				
285768	LOC285768	-0.44	0.046	100505598	PEX5L-AS2	-0.45	0.042				
286059	LOC286059	0.54	0.011	100505644	ELFN1-AS1	0.57	0.008				
286083	LOC286083	0.55	0.009	100505650	LOC100505650	-0.57	0.007				
286101	ZNF252P	-0.44	0.048	100505735	LOC100505735	0.49	0.024				
286149	LOC286149	0.63	0.002	100505760	LOC100505760	0.46	0.036				
317648	NOP14-AS1	-0.45	0.039	100505828	LOC100505828	0.47	0.033				
337970	KRTAP19-3	0.53	0.013	100505839	SH3PXD2A-AS1	0.51	0.017				
338321	NLRP9	0.50	0.021	100505877	UBE2E2-AS1	-0.49	0.024				
339448	C1orf174	0.44	0.047	100505946	LOC100505946	0.44	0.046				
339665	SLC35E4	-0.53	0.013	100506059	LOC100506059	0.44	0.043				
340526	RGAG4	0.46	0.035	100506161	TAF1A-AS1	-0.57	0.006				
342574	KRT27	0.49	0.025	100506189	LOC100506189	0.49	0.023				
344595	LINC00883	-0.46	0.037	100506327	LINC01128	0.53	0.014				
346171	ZFP57	-0.44	0.044	100506530	LOC100506530	0.56	0.009				
347744	C6orf52	-0.44	0.048	100506591	LOC100506591	-0.70	0.001				
353299	RGSL1	0.45	0.042	100506813	LOC100506813	0.51	0.018				
353500	BMP8A	-0.43	0.050	100507027	LINC00948	0.48	0.028				
374393	FAM111B	-0.51	0.019	100507064	TEX26-AS1	0.44	0.047				
378832	COL18A1-AS1	0.50	0.021	100507360	LOC100507360	-0.62	0.002				
386618	KCTD4	0.49	0.023	100507557	LOC100507557	-0.46	0.036				

Table S4: Table of the number of OTUs per bacterial family that was either up- or down regulated in IBS volunteers relative to HCs.

Down Regulated in IBS		Up Regulated in IBS	
Family	# of OTUs	Family	# of OTUs
Prevotellaceae	9	Lachnospiraceae	25
RikenellaceaeII	5	RikenellaceaeII	8
Lachnospiraceae	3	unclassified	8
Peptostreptococcaceae	1	Pseudomonadaceae	7
Peptococcaceae	1	Veillonellaceae	5
Spirochaetaceae	1	Enterobacteriaceae	2
Dethiosulfovibrionaceae	1	Carnobacteriaceae	2
		Bacillaceae	2
		Streptococcaceae	1
		Burkholderiaceae	1
		Pasteurellaceae	1
		Enterococcaceae	1
		Ruminococcaceae	1
		Microbacteriaceae	1

Table S5: Table of the number of OTUs per bacterial family that was either up- or down regulated in IBS-D volunteers relative to HCs.

Up Regulated in IBS		Down Regulated in IBS	
Family	# of OTUs	Family	# of OTUs
RikenellaceaeII	3	Lachnospiraceae	18
Burkholderiaceae	2	unclassified	8
Lachnospiraceae	2	Streptococcaceae	4
unclassified	1	Propionibacteriaceae	4
Prevotellaceae	1	Pseudomonadaceae	3
Peptococcaceae	1	Micrococcaceae	3
Bifidobacteriaceae	1	RikenellaceaeII	2
Lactobacillaceae	1	Carnobacteriaceae	2
Comamonadaceae	1	Bacillaceae	2
Halomonadaceae	1	Microbacteriaceae	2
Moraxellaceae	1	Bacteroidaceae	2
		Burkholderiaceae	1
		Enterobacteriaceae	1
		Enterococcaceae	1
		Ruminococcaceae	1
		Mycoplasmataceae	1
		Planctomycetaceae	1
		Gemellaceae	1
		Anaerolinaceae	1
		Jonesiaceae	1
		Nitrospiraceae	1
		Campylobacteraceae	1
		Staphylococcaceae	1
		Cellulomonadaceae	1
		Coriobacteriaceae	1
		Neisseriaceae	1
		Corynebacteriaceae	1

Table S6: Table of the number of OTUs per bacterial family that was either up- or down regulated in IBS-D volunteers relative to HCs.

Up Regulated in IBS		Down Regulated in IBS	
Family	# of OTUs	Family	# of OTUs
Veillonellaceae	6	Lachnospiraceae	4
unclassified	1	unclassified	3
Spirochaetaceae	1	Veillonellaceae	2
Prevotellaceae	1	Peptococcaceae	2
Porphyromonadaceae	1	Corynebacteriaceae	2
Peptococcaceae	1	Streptococcaceae	1
Lachnospiraceae	1	Enterobacteriaceae	1
		Chromatiaceae	1

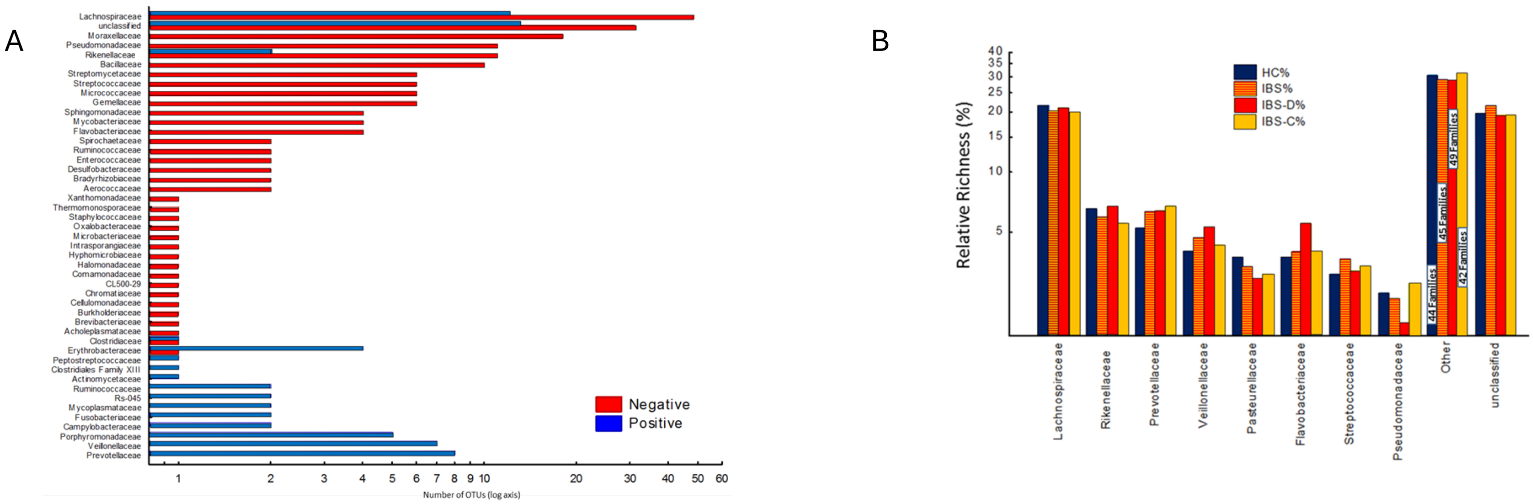

Supplemental Figure 1: Differential species abundance in IVP and participant groups. (A) Correlation plot of OTU and severity of IVP. The number of OTU (x-axis) per bacterial family (y-axis) that were significantly ($r^2 > 0.22$; $p < 0.05$), negatively (red bars) and positively (blue bars) correlated to severity of the induced visceral pain (IVP) was visually represented. The severity of IVP negatively correlated to most Lachnospiraceae and Rikenellaceae and all Moraxellaceae, Pseudomonodaceae, Bacillaceae. Prevotellaceae and Veillonellaceae showed positive correlation to the severity of IVP. (B) Relative bacterial richness by participant group. Bacterial families are represented on the x-axis and the relative richness (expressed as a %) of each microbial family for healthy controls and IBS as a group (consisting of IBS-D and IBS-C and IBS-M) and in IBS-D and in IBS-C separately are expressed on the y-axis. The Prevotellaceae, Veillonellaceae, Flavobacteriaceae, and Streptococaceae show greater OTU richness in IBS and IBS-subtypes than in HCs, whereas the Lachnospiraceae and Pasteurellaceae show consistent decreased richness in all sub-groups of individuals who experience chronic visceral hypersensitivity.

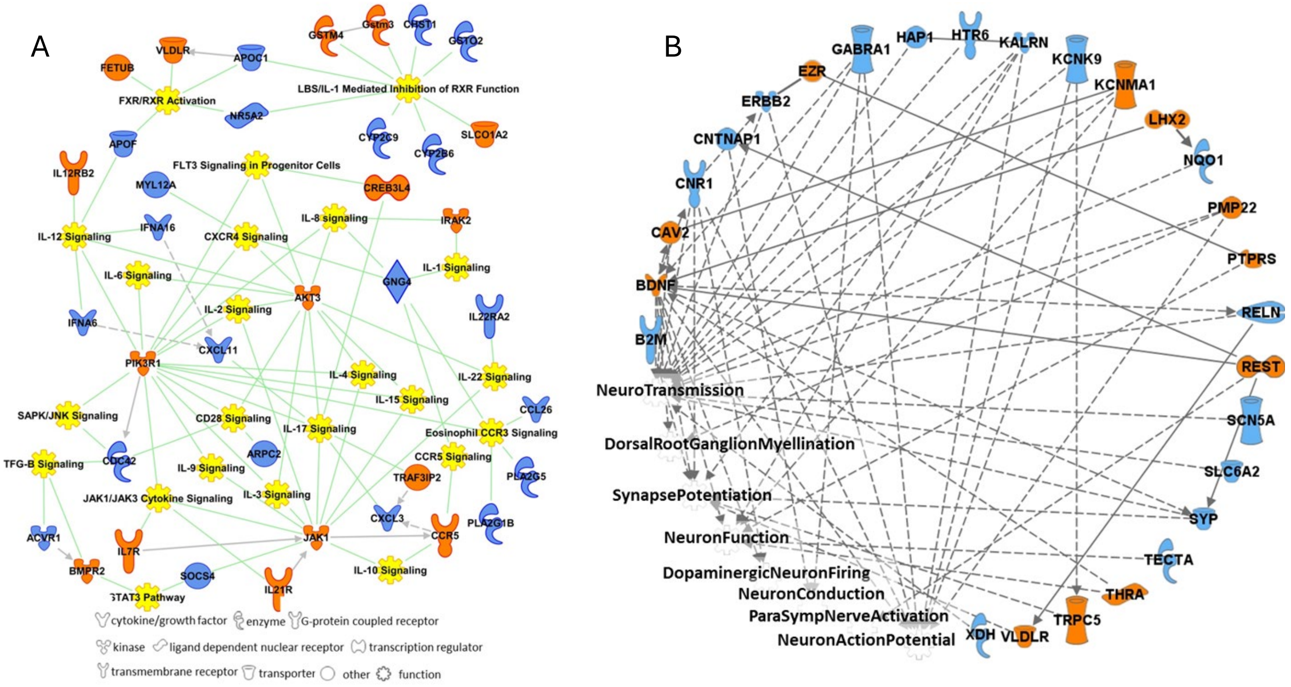

Supplemental Figure 2: Functional Linkage Networks. (A) Functional linkage network of genes related to cytokine signaling and correlated to IVP. Genes in orange are negatively and genes blue are positively correlated to IVP. All genes in the network are also correlated to bacterial expression. Lines connecting genes and functions denote indirect (dotted lines) and direct (solid lines) relationships. (B) Functional linkage network of genes related to neuronal and neuronal-supportive processes.
